# Saline is a more appropriate solution for microvesicles for flow cytometric analyses

**DOI:** 10.18632/oncotarget.15987

**Published:** 2017-03-07

**Authors:** Xing Xin, Peiling Zhang, Xing Fu, Xia Mao, Fankai Meng, Ming Tian, Xiaojian Zhu, Hanying Sun, Li Meng, Jianfeng Zhou

**Affiliations:** ^1^ Department of Hematology, Tongji Hospital, Tongji Medical College, Huazhong University of Science and Technology, Wuhan 430030, P. R. China

**Keywords:** phosphate-buffered saline, saline, flow cytometry, microvesicles

## Abstract

Microvesicles (MVs) are carriers of molecular and oncogenic signatures present in subsets of tumor cells and tumor-associated stroma, and a focus of cancer research. Although methods to detect MVs are mature, we were concerned that the buffer used could lead to false results when quantitating MVs by flow cytometry. In this work, we detected MVs by flow cytometry withthree different solutions: water, saline, and phosphate-buffered saline (PBS). The results demonstrated that PBS, when reacted with annexin V binding buffer, produced nano-sized vesicles even when there were no MVs in the sample. No similar events occurred in the saline and water groups (*P* < 0.01). Annexin V positive rate increased significantly when PBS was used as the buffer, compared to saline and water. These false negative results were also observed when we quantified some markers of MVs such as CD3 and CD19. A probable explanation for these findings is the production of insoluble Ca(H_2_PO_4_)_2_ or Ca_3_PO_4_ from calcium in the binding buffer and phosphate in PBS. Thus, considering the osmotic pressure of water, we suggest that saline is a more suitable buffer when counting MVs by flow cytometry.

## INTRODUCTION

Cellular microvesicles (MVs), 0.1–1 μm in size, are released by various cell types, especially cancer cells that are undergoing stress and activation [1–3]. During recent decades, extensive research revealed that MVs carry their parental cell proteins, lipids, and nucleic acids, which may be transferred between cells [4–6]. MV-mediated cargo transfer to adjacent or remote cells affects tumor progression and provides a potential source of disease-related biomarkers [7]. Multiple studies have demonstrated that tumor cells may locate to undetectable sites but their MVs circulate in the blood, transporting information about the cancer [1–3]. Although the understanding of MV biology remains a major challenge, their characteristics create new opportunities for advances in cancer diagnostics and therapeutics. Such information suggests the possibility of using MVs in biological fluids as markers of cancer pathology, as more feasible “liquid-biopsy” material to gain diagnostic information, and to follow disease progression and the response to clinical treatment through a simple blood test or cerebrospinal fluid collection [1, 3, 7–9].

Uniform methods to isolate and identify MVs are not yet defined. Electron microscopy, atomic force microscopy, nanoparticle tracking analysis, and flow cytometry have been reported in many studies [10]. Among all, flow cytometry is effective for high-throughput quantification and multiparameter characterization of MVs [11–14]. Most researchers have confirmed the presence of MVs by assessing annexin V-positive vesicles using phosphate-buffered saline (PBS). However, we believe that using the annexin V binding buffer with PBS could generate numerous nano-sized vesicles that would seriously affect quantification of these structures. The aim of the current study was to investigate whether mixing the annexin V binding buffer with PBS modified the flow cytometry results.

## RESULTS

### Detection of MVs by electron and fluorescence microscopy

**Figure 1:**
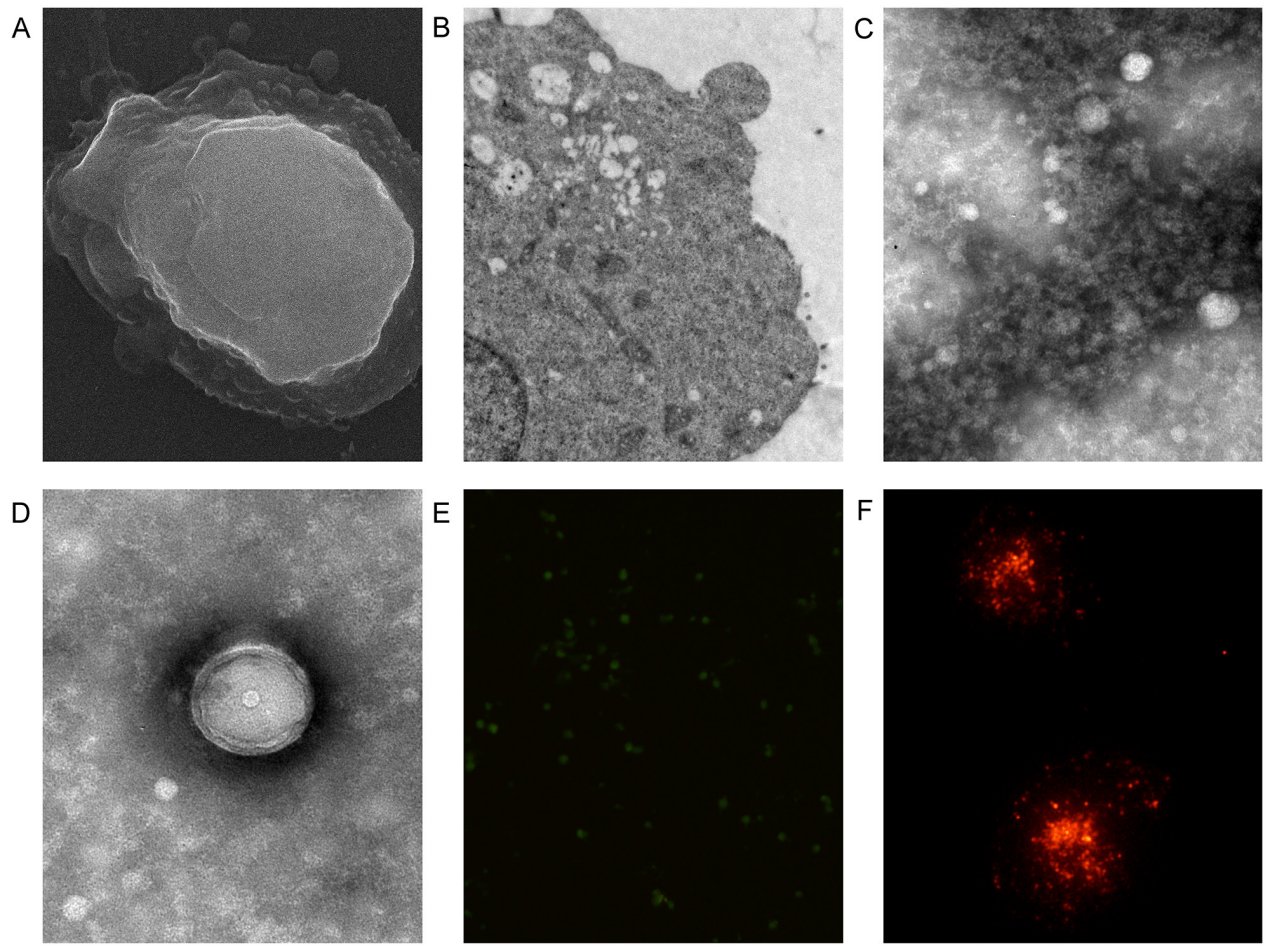
Fluorescent labeling and electron microscopy of microvesicles (MVs) K562 cells were observed by scanning **(A)** and transmission **(B)** electron microscopy. MVs were observed by transmission electron microscopy **(C, D)**. **(E)** MVs were labeled directly by PKH67. **(F)** MVs labeled by PKH26 were devoured by human umbilical vein endothelial cells after 12 h.

K562 cells were observed by scanning electron microscopy (SEM) and transmission electron microscopy (TEM) (Figure 1A, 1B). Extracellular vesicles were visible on the surface of K562 cells through SEM (Figure 1A). The MV pellet was observed by TEM and they exhibited circular structures of different sizes with a bilayer (Figure 1C, 1D). Most vesicles were less than 1 μm in diameter. PKH67-labeled MVs were observed directly by fluorescence microscopy (Figure 1E). PKH26-labeled MVs were devoured by human umbilical vein endothelial cells (HUVECs) after 12 h (Figure 1F).

### Annexin V binding buffer mixed with PBS generated nano-sized vesicles

**Figure 2:**
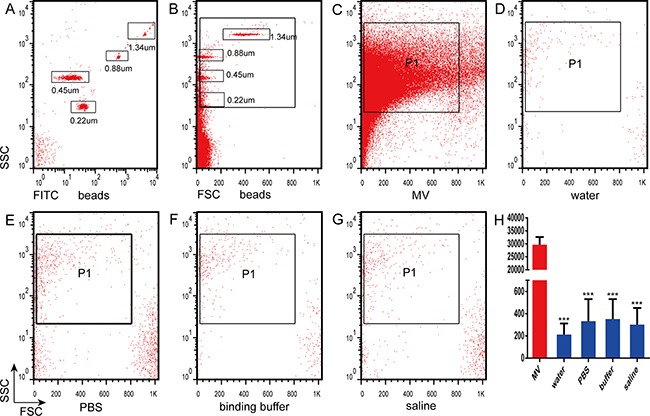
Analysis of nanoparticles by flow cytometry **(A)** Nanoparticles were analyzed by flow cytometry based on fluorescein isothiocyanate fluorescence and side scatter count (SSC). **(B)** Fluorescent nanoparticles were analyzed by flow cytometry according to the SSC and forward scatter count (FSC). Nanoparticle counts were determined by flow cytometry in **(C)** microvesicles (MVs), (D) water (H_2_O), **(E)** phosphate-buffered saline (PBS), **(F)** annexin V binding buffer (binding buffer), and **(G)** saline. **(H)** Quantitation of the counts in C-G.

Initially, we discriminated sizes by flow cytometry using fluorescent microbeads of 0.22, 0.45, 0.88, and 1.34-μm diameters. The size position of the MV gate was assessed in forward versus side scatter dot plots (Figure 2A, 2B). Flow cytometric analysis demonstrated that the majority of MVs were smaller than the 1.34-μm beads. All gating events were also analyzed for phosphatidylserine (PS) by studying annexin V binding to distinguish true events (annexin V positive) from background noise. To detect nano-sized vesicles generated by mixing the annexin V binding buffer with PBS using flow cytometry, we used blank control groups containing water, saline, and PBS. To exclude pre-existing nano-sized vesicles from the blank and annexin V binding buffer solutions, we initially double-filtered the solutions through a 0.22-μm filter and then determined MV-sized vesicle counts by flow cytometry. MVs from K562 cells were analyzed at the same time as a positive control. The number of events recorded during 30 s by flow cytometry were as follows: 30,120 ± 3041 for MVs isolated from K562 cells, 225 ± 101 for water, 340 ± 197 for PBS, 363 ± 181 for annexin V binding buffer, and 308 ± 151 for saline (*P* < 0.0001 for K562 cells vs. all other groups) (Figure 2C–2H).

**Figure 3:**
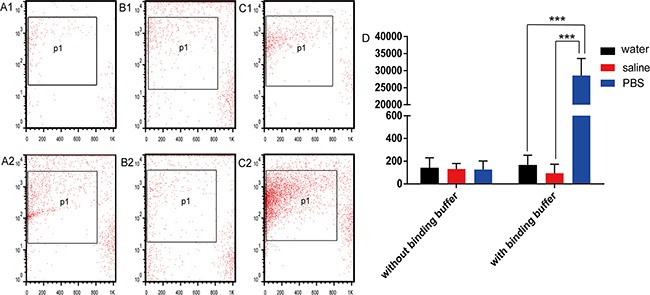
Generation of nanovesicles with and without annexin V binding buffer Nanovesicles generated by **(A1)** water, **(B1)** saline, and **(C1)** phosphate-buffered saline (PBS) without annexin V binding buffer were analyzed by flow cytometry. Nanovesicles generated by **(A2)** water, **(B2)** saline, and **(C2)** PBS with annexin V binding buffer were analyzed by flow cytometry. **(D)** Quantitation of the flow cytometry results.

For further confirmation that PBS mixed with annexin V binding buffer could generate nano-sized vesicles, water, saline, and PBS were mixed with annexin V binding buffer. The counts of nano-sized vesicles from each group were 140 ± 92 (water), 165 ± 87 (water with annexin V binding buffer), 131 ± 50 (saline), 93 ± 79 (saline with annexin V binding buffer), 126 ± 76 (PBS), and 28,551 ± 5010 (PBS with annexin V binding buffer). The counts of nano-sized vesicles in tubes of PBS with annexin V binding buffer were significantly different (n = 5, *P* < 0.0001). There was no significant difference between the other groups (n = 5, *P* = 0.3310, and *P* = 0.4229, respectively) (Figure 3).

**Figure 4:**
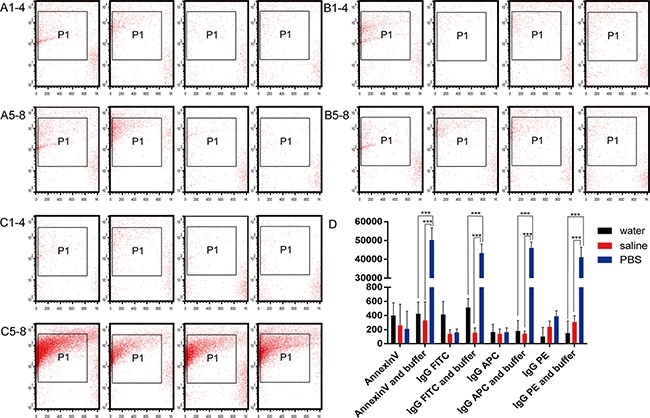
Effects of isotype control antibodies on nanovesicle counts Nanovesicle counts without annexin V binding buffer in **(A1-4)** water, **(B1-4)** saline, and **(C1-4)** phosphate-buffered saline (PBS) were determined by flow cytometry in the presence of antibodies to annexin V, IgG1-FITC, IgG1-APC, and IgG1-PE (left to right panels, respectively). Nanovesicle counts with annexin V binding buffer in **(A5-8)** water, **(B5-8)** saline, and **(C5-8)** PBS were determined by flow cytometry in the presence of antibodies to annexin V, IgG1-FITC, IgG1-APC, and IgG1-PE (left to right panels, respectively). **(D)** Quantitation of the nanovesicle results in the three different solutions with four different antibodies, with and without annexin V binding buffer.

To investigate whether the nano-sized vesicles affected the results of surface labeling on MVs, we used isotype-control IgG1-fluorescein isothiocyanate (FITC), -allophycocyanin (APC), -phycoerythrin (PE), and annexin V antibodies. The results were similar with all antibodies when counting nano-sized vesicles in water (n = 5, *P* > 0.05, Figure 4A). The counts of nano-sized vesicles in the saline group were 138 ± 60 and 156 ± 69, 138 ± 70 and 142 ± 36, 238 ± 82 and 307 ± 90, and 260 ± 300 and 330 ± 265 with the IgG1-FITC, -APC, -PE, and annexin V antibodies, without and with annexin V binding buffer, respectively (n = 5, *P* > 0.05, Figure 4B). The numbers of nano-sized vesicles in the PBS group were 159 ± 50 and 43,291 ± 4920, 162 ± 63 and 46,012 ± 3200, 389 ± 79 and 41,088 ± 5360, and 210 ± 250 and 50,190 ± 6548 in the IgG1-FITC, -APC, -PE, and annexin V antibodies, without and with annexin V binding buffer, respectively (n = 5, *P* < 0.0001, Figure 4C, 4D).

### Annexin V binding buffer mixed with PBS generated nano-sized vesicles and increased the false positive results when analyzed by flow cytometry

**Figure 5:**
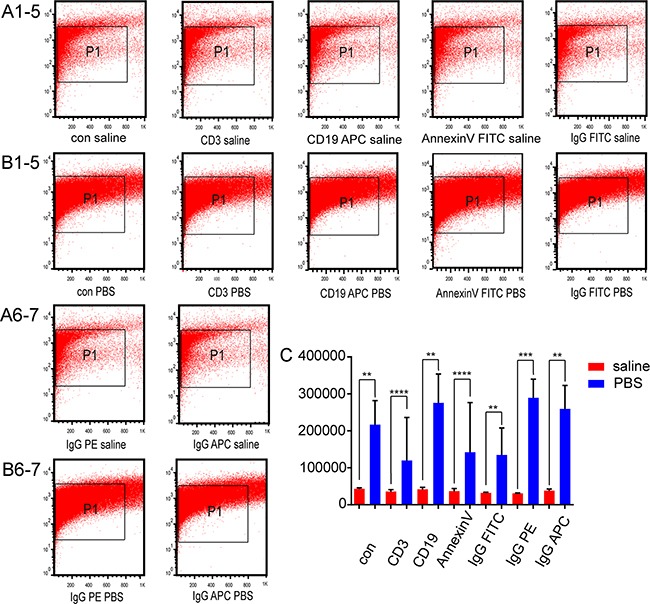
Effects of CD3, CD19, annexin V, and isotype control antibodies on nanovesicle counts Nanovesicle counts generated with CD3, CD19, annexin V, and isotype control antibodies with annexin V binding buffer in **(A1-7)** saline and **(B1-7)** phosphate-buffered saline (PBS) were analyzed by flow cytometry. All numbering is left to right. **(C)** The quantitation of nanovesicle counts was determined by flow cytometry in saline and PBS with annexin V binding buffer.

**Figure 6:**
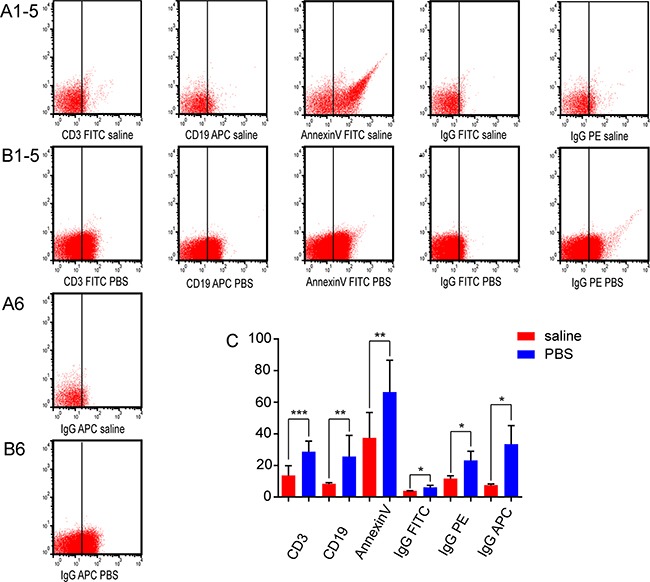
Effects of CD3, CD19, annexin V, and isotype control antibodies on the nanovesicle positive rate CD3, CD19, annexin V, and isotype control positive rates with annexin V binding buffer in **(A1-6)** saline and **(B1-6)** PBS were analyzed by flow cytometry. All numbering is left to right. **(C)** The positive rates were analyzed by flow cytometry in saline and PBS with annexin V binding buffer.

For analysis of the nano-sized vesicles generated by mixing PBS with annexin V binding buffer, comparisons were made to MVs isolated from K562 cells. We selected the CD3-FITC, CD19-APC, annexin V-FITC, IgG1-FITC, IgG1-PE, and IgG1-APC antibodies to stain the MVs. Annexin V binding buffer was added to every sample. Nano-sized vesicles in the PBS group were significantly elevated compared to the saline group (Figure 5) (Supplementary Table 1). The positive rates in PBS group were also increased compared to the saline group (Figure 6) (Supplementary Table 1).

### Nano-sized vesicles and positive rates in different solutions following drug-induced apoptosis in K562 cells analyzed by flow cytometry

**Figure 7:**
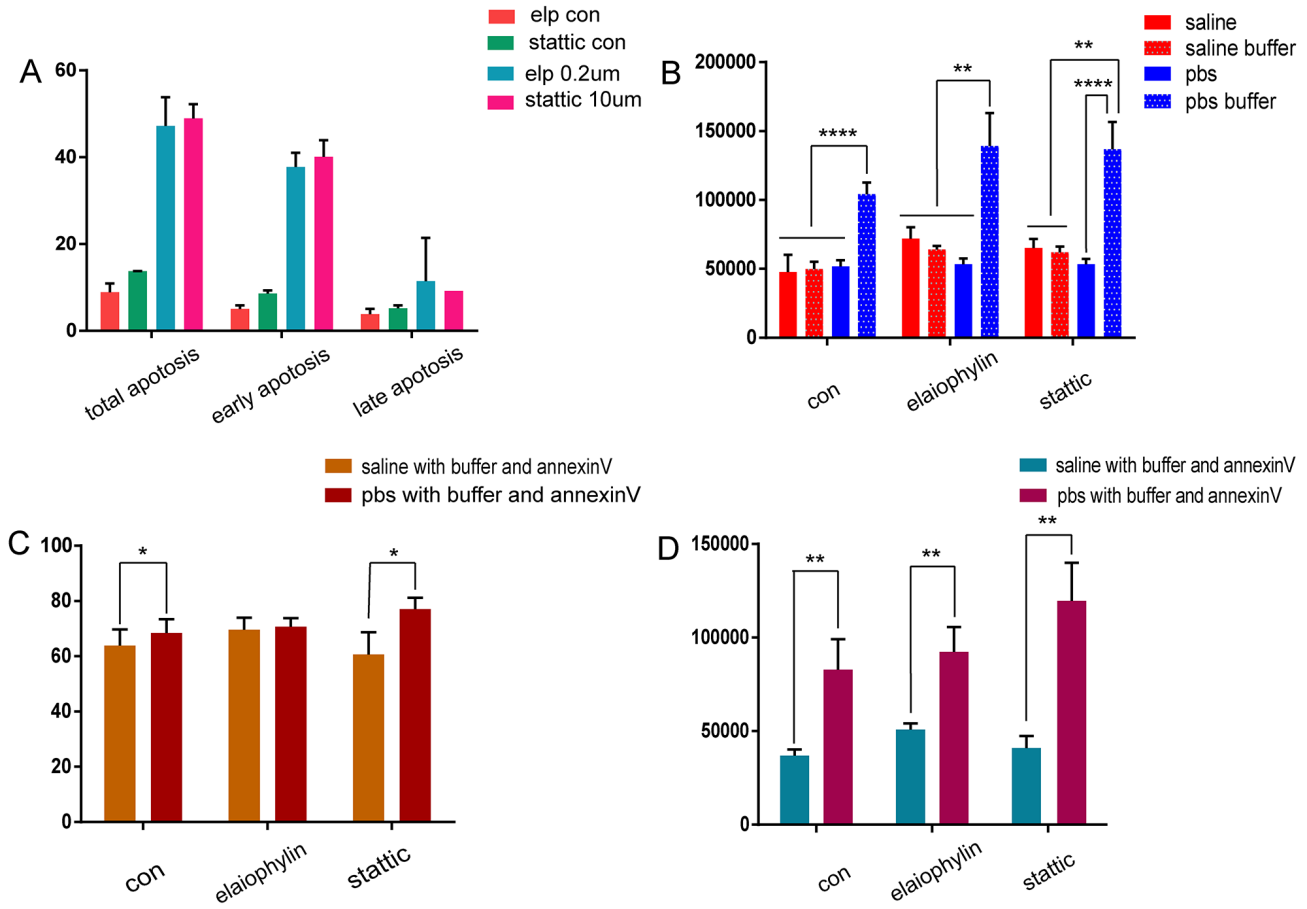
Annexin V positive rates and nano-sized vesicle counts in different solutions following drug-induced apoptosis in K562 cells analyzed by flow cytometry **(A)** Flow cytometry analysis of apoptosis induced by elaiophylin and stattic in K562 cells. **(B)** Nanovesicle counts, **(C)** annexin V positive rates, and (D) annexin V positive nanovesicle counts generated from the drug-induced apoptosis of K562 cell with annexin V binding buffer in saline or PBS.

The autophagy inhibitor elaiophylin (0.2 μM) and the STAT3 inhibitor stattic (10 μM) were used to induce apoptosis in K562 cells. After 24 h in serum-free medium, the percentages of total, early, and late apoptosis of K562 cells were 47.2 ± 6.69, 48.9 ± 3.30, and 35.8 ± 3.32%, and 40.1 ± 3.81, 11.4 ± 10.0, and 9.17 ± 0.01% for elaiophylin and stattic, respectively (n = 3). The total, early, and late apoptosis percentages of control K562 cells (without elaiophylin and stattic) in serum-free medium for 24 h were 8.86 ± 2.05%, 13.7 ± 0.07%, 5.01 ± 0.85%, 8.55 ± 0.79%, 3.85 ± 1.20%, and 5.18 ± 0.72%, respectively (n = 3) (Figure 7A).

Saline and PBS were used with the control, elaiophylin, and stattic groups. Equal numbers of MVs from the same group were dissolved in saline or PBS, with and without binding buffer. The counts of nano-sized vesicles in tubes containing PBS with binding buffer were significantly greater than the corresponding saline group (n = 5, *P* < 0.01) (Figure 7B) (Supplementary Table 2).

For analysis of the positive rate and the positive nano-sized counts, the control, elaiophylin, and stattic groups were dissolved in saline or PBS with binding buffer and annexin V antibody. The positive percentages were 63.8 ± 5.83%, 68.5 ± 4.93%, 69.6 ± 4.32%, 70.7 ± 3.11%, 60.6 ± 8.06%, and 77.1 ± 4.12%, respectively (Figure 7C). The positive nano-sized counts in the control, elaiophylin, and stattic groups dissolved in saline or PBS with binding buffer and annexin V antibody were 36,953 ± 3221, 82,849 ± 16383, 50,840 ± 3371, 92,317 ± 13,404, 40,886 ± 6557, and 119,556 ± 20,375, respectively (Figure 7D) (Supplementary Table 3).

## DISCUSSION

Intercellular communication is essential for cancer cells to survive and thrive. Extracellular vesicles such as exosomes, MVs, and large oncosomes are involved in this communication process by shuttling reciprocal signals and other molecules between cancer and stromal cells, including fibroblast, endothelial, and immune cells [15–17]. Extracellular vesicles could act as a platform for liquid biopsy in tumors, such as glioblastoma [3, 7, 18, 19]]. In addition, increased numbers of MVs and the expression of specific MV markers such as CD19 in chronic lymphocytic leukemia are associated with disease progression and malignancy [20]. Many studies have shown that increased circulating MVs indicate poor prognosis and disease progression in some cancers [20]. Thus, precise enumeration of MVs is very important in this area.

MVs are defined as particles between 100 and 1000 nm in diameter that typically exhibit PS on the outer leaflet of their plasma membranes. Most studies have shown that MVs are typed and identified by fluorescence-activated cell sorting. Because exposure of PS is a typical marker for MVs, the standard procedure is to stain PS exposed on the external surface with annexin V [21–23]. Apoptotic bodies are also derived from the membrane of parental cells, with PS exposed on the surface. As a result, the methods of detection are similar for MVs and apoptotic bodies. Annexin V binding to PS is calcium-dependent [24], which leads to a lack of sensitivity and specificity when used to define MV populations [23, 25]. When using annexin V to define MVs, calcium-phosphate microprecipitates are observed in the MV gate and they increase the non-specific binding of annexin V, potentially leading to false-positive detection of MVs. Reporting increased MV-like microprecipitates might provide clinicians with erroneous information and misdiagnosis of diseases. Larson et al. designed an experiment showing that PBS generated an increasing amount of calcium-phosphate microprecipitates with increasing concentrations of CaCl_2_. Similar to our findings, they also found that the median fluorescence signal intensity increased with increasing CaCl_2_ concentrations when adding fluorescently labeled antibodies to the microprecipitates [22].

In our study, we confirmed that annexin V binding buffer contained Ca^2+^ and, when added to PBS, generated microprecipitates that led to false positive results. Thus, PBS was not suitable for MV counting. Resuspending the MV pellet in 100 μL of saline could avoid artifacts from the calcium-phosphate microprecipitates, and false-positive results. Saline appears to be a better choice to improve the sensitivity of MV-based lipid biopsy and provides important information about optimal molecular monitoring schedules in cancer diagnosis and evaluating the efficacy of different treatments. Exosomes, with a diameter of 100 nm, could not be detected by flow cytometry because the lower limit of detection was 300 nm. Thus, we consider that PBS will not affect exosome counts.

## MATERIALS AND METHODS

### Cell culture and isolation of MVs

The human chronic myeloid leukemia blast crisis cell line, K562, was purchased from the American Type Culture Collection (Manassas, VA, USA). HUVECs were obtained from the cell bank of the Chinese Academy of Sciences. K562 cells were cultured in RPMI 1640 medium containing 10% fetal bovine serum at 37°C in 5% CO_2_. Before isolation of MVs, K562 cells were adjusted to 1 × 10^6^/mL and cultured in serum-free RPMI 1640 for 4 h. Cells were pelleted at 100 × *g* for 5 min followed by centrifuging the supernatant at 3000 × *g* for 10 min to remove cellular debris. The resultant supernatant was centrifuged at 5000 × *g* for 30 min to remove smaller cellular debris, followed by centrifugation at 16,000 × *g* for 90 min to obtain MVs [26]. HUVECs were cultured in DMEM medium containing 10% fetal bovine serum at 37°C in 5% CO_2_.

### Labeling of MVs with PKH26 and PKH67

MVs were labeled with PKH26 red and PKH67 green fluorescent cell linker mini kits (Sigma-Aldrich, St. Louis, MO, USA) following the manufacturer's instructions. MVs were visualized under a fluorescence microscope.

### Antibodies and reagents

Monoclonal antibodies (CD3-FITC, CD19-APC, and annexin V-FITC) were purchased from BD Biosciences (Franklin Lakes, NJ, USA). Appropriate APC, PE, and FITC isotype antibodies were used as negative controls (BD Biosciences). The fluorescent nanoparticle size standard kit was purchased from Spherotech (Lake Forest, IL, USA; Cat. No. NFPPS-52-4K). Elaiophylin was provided by North China Pharmaceutical Group Corporation New Drug R&D center. Stattic was purchased from Sigma-Aldrich.

### Labeling of MVs and flow cytometric analyses

All analyses were performed on an LSR II flow cytometer (BD Biosciences) with DIVA (BD Biosciences) and FlowJo software [17]. The MV gate was established based on forward and side scatters [27] using fluorescent microbeads of 0.22, 0.45, 0.88, and 1.34 μm diameters, and defining MVs as events less than 1.34 μm. The lower detection limit as a threshold above the electronic noise of the flow cytometer was 0.3 μm [14]. To reduce background event numbers that affected the experimental results, the PBS, saline (0.9% w/v NaCl), and annexin V binding buffers were double filtered through a 0.22-μm filter (EMD Millipore, Billerica, MA, USA) and stored at 4°C. Two groups of MVs were isolated from 40 mL of culture medium containing 1 × 10^6^/mL of K562 cells, and diluted in 1 mL of double filtered saline or PBS. MVs were suspended by agitating for 5 s in a whirlpool oscillator, and a 100-μL sample was aspirated into different flow tubes.

Two blank groups were designed using fluorescent nanoparticles to confirm the MV gate, each with three subgroups: double filtered water, saline, and PBS without MVs. The volume of each tube was 100 μL. To determine whether PBS mixed with annexin V binding buffer generated nano-sized vesicles, the annexin V antibody was only added to tubes without the 10× annexin V binding buffer. In another blank group, the annexin V isotype-matched control IgG1-FITC, IgG1-APC, and IgG1-PE antibodies were added at 10, 6, 5, and 5 μL, respectively, with and without annexin V binding buffer. In the MV group, 6 μL of CD3, 5 μL of CD19, 6 μL of IgG1-FITC, 5 μL of IgG1-APC, 5 μL of IgG1-PE, and 10 μL of annexin V antibodies were added into the same solutions (saline and PBS) with and without annexin V binding buffer. All antibodies added to the MV and blank groups were incubated for 30 min at room temperature in the dark. Event numbers of equal sample volumes were counted for 30 s.

### Statistical analyses

All statistical analyses were carried out using GraphPad Prism version 6.0 (GraphPad, La Jolla, CA, USA) and SPSS for Windows, version 17.0 (IBM, Chicago, IL, USA). Non-parametric and unpaired t-test comparisons were used to compare groups; the rates between groups were compared by the chi-square test. Two-sided *P* < 0.05 was defined as being statistically significant.

## SUPPLEMENTARY MATERIALS FIGURES AND TABLES


