# Supplementary material for: Saline is a more appropriate solution for microvesicles for flow cytometric analyses
Source: Oncotarget. 2017 Mar 7;8(21):34576–85. doi: 10.18632/oncotarget.15987 (PMC5470992; doi:10.18632/oncotarget.15987)
Supplement: Supplementary file 1 [file oncotarget-08-34576-s001.pdf]

Saline is a more appropriate solution for microvesicles for flow cytometric analyses

SUPPLEMENTARY TABLES

Supplementary Table 1: Annexin V binding buffer mixed with different solutions generated nano-sized vesicles and positive results when analyzed by flow cytometry

	nano-sized vesicle counts (means ± SD)		*P*	positive rate (%)		*P*
	saline	PBS		saline	PBS	
CD3	35,610 ± 5281	119,517 ± 116,707	*P* < 0.0001	13.7 ± 6.1	28.7 ± 6.7	*P* = 0.0004
CD19	41,458 ± 5785	275,491 ± 78,459	*P* = 0.0060	8.35 ± 0.81	25.6 ± 13.5	*P* = 0.0072
AnnexinV	36,540 ± 7163	141,963 ± 134,504	*P* < 0.0001	37.5 ± 16.0	66.5 ± 20.1	*P* = 0.0066
IgG1-FITC	31,952 ± 180	134,348 ± 73,553	*P* = 0.0012	3.98 ± 0.07	6.21 ± 1.24	*P* = 0.0359
IgG1-PE	30,552 ± 1276	289,326 ± 50,395	*P* = 0.0009	11.7 ± 1.76	23.2 ± 5.73	*P* = 0.0291
IgG1-APC	37,914 ± 4750	259,049 ± 63,672	*P* = 0.0039	7.54 ± 0.73	33.5 ± 11.8	*P* = 0.0191

Supplementary Table 2: Nano-sized vesicle counts in different solutions following drug-induced apoptosis in K562 cells analyzed by flow cytometry

	nano-sized vesicle counts (means ± SD)					
	saline	saline plus binding buffer	PBS	(VS)	PBS plus binding buffer	*P*
Control	47,806 ± 12,387	49,995 ± 5311	51,736 ± 4436		104,212 ± 8617	*P* < 0.0001
Elaiophylin	71,975 ± 8306	64,003 ± 2829	53,406 ± 4235		139,258 ± 23,882	*P* = 0.009, *P*=0.003, *P* = 0.002
Stattic	65,202 ± 6616	62,025 ± 4286	53,469 ± 3747		136,995 ± 19,544	*P* = 0.001, *P* = 0.001, *P* < 0.0001

Supplementary Table 3: Annexin V positive rates and nano-sized vesicle counts in different solutions following drug-induced apoptosis in K562 cells analyzed by flow cytometry

	Annexin V positive rate (%)			Annexin V positive nano-sized vesicle counts		
	saline plus binding buffer	PBS plus binding buffer	*P*	saline plus binding buffer	PBS plus binding buffer	*P*
control	63.8 ± 5.83	70.7 ± 3.11	*P* = 0.030	36,953 ± 3221	82,849 ± 16,383	*P* = 0.002
elaiophylin	68.5 ± 4.93	60.6 ± 8.06	*P* = 0.671	50,840 ± 3771	92,317 ± 13,404	*P* = 0.005
stattic	69.6 ± 4.32	77.1 ± 4.12	*P* = 0.017	40,886 ± 6557	119,556 ± 20,375	*P* = 0.003
